# Diphtheria Treated with Carbolic Acid

**Published:** 1873-11

**Authors:** C. G. Rothe

**Affiliations:** Altenburg, Germany


### DIPHTHERIA TREATED WITH CARBOLIC ACID.

BY DR. C. G. ROTHE, ALTENBURG, GERMANY.

My first trials of the local treatment of Diphtheria by Carbolic Acid, were made in the fall of 1869, and an account thereof published the following June, in the *Berliner Klinische Wochenschrift.* Since that time various other communications on this subject have appeared in medical periodicals, the writers differing considerably in the results of their experience. As none of these gentleman make any allusion to my previous experiments in this line, it is to be supposed that, with each one of them, the application of the remedy to this disease was an original suggestion. I do not consider that any special credit attaches to a man because he happens to be the first to apply a remedy, whose qualities are well known, to a given disease, especially when such application would *a priori* appear to be particularly indicated. But I am surprised that some of the gentlemen who have written on this subject should be so little conversant with the periodical medical literature of the day, as to suppose that their use of carbolic acid in the treatment of diphtheria was the first instance of the kind on record.

The object of this paper, however, is not to vindicate any claim of mine to priority in the use of the treatment, (which I consider a matter of no consequence), but to call attention once more to the value thereof, especially in view of the difficulties and discouragements that have been experienced by some of those who have tried it. Although some of the experiments with this method have attained admirable results, (Dr. Schlier in Bavaria, Dr. Helfer in Leipzig, and others), yet the majority, (among them Dr. Letzerich and Dr. Lowinson), consider the action of carbolic acid as, on the whole, not superior to that of other escharotics, previously in use; while a few (Jas. E. Reeves and Dr. G. Hill) are directly opposed to its use, as being, in their opinion, either valueless or injurious.

Now, I have treated one hundred and fifty cases of diphtheria in this manner since 1869, *all of which have recovered.* Whence then this difference in results? Is it due to different degrees of severity in the cases treated, or to a difference in the preparation used and the method of its application?

In my own experiments, I carefully excluded all such attacks of pharyngeal and tonsillar catarrh, or ulceration, as were not marked by the high fever, the severity of the local implecation, characteristic of diphtheria. A considerable number of them, too, especially those complicated with scarlatina (during an epidemic) left nothing to be desired in the way of severity; so much so, indeed, that, in spite of my previous good fortune, I despaired of their recovery for days, and even weeks. One condition of things, however, never re-appeared in my practice, during all this time, that is, the descent of the exudation into the larynx below the vocal chords, giving rise to the symptoms of croup; although hoarseness and even aphonia gave evidence of the implication of the upper part of the larynx. Inasmuch as this fatal complication had not been at all rare in my experience of diphtheria previous to 1869, I considered myself justified in attributing its absence, not to any difference in the character or severity of the disease, but to early and thorough use of the remedy on which I now relied. In only one case, to which I was not called till the seventh day, did I find, besides the characteristic exudation on the soft palate and tonsils, all the evidences of stenosis of the larynx. Fortunately the patient was not an infant, but a sturdy boy eleven years old, who, under the application of frequent pencillings to the glottis, recovered in eight days.

It appears, then, that the difference between my results and those of other observers must depend upon a difference in the preparation used or in the method of its application: perhaps in both.

From the time of my very earliest experiments, I have been in the habit of using a very concentrated solution, viz: one part of the pure crystalized carbolic acid to seven parts of the solvent; a much stronger preparation than I find is used by others.

I first dissolve one pint of Acid with one pint of Alcohol, for the purpose of securing the antiseptic effect of the latter, and then add five pounds of water; finally, both to aid in counteracting the disagreeable odor of the acid and to heighten the antiseptic qualities of the mixture, I add one pint of tincture of Iodine. This addition of the Iodine was, at first, more accidental than designed. I had used Carbolic Acid inhalations for consumptives as early as the summer of 1869, and had added the iodine, partly in view ot Piorry's good results with Iodine inhalations, and partly to correct the odor of the acid. I had never thought of any chemical transformation being produced. During the ensuing year I received a pamphlet from Errico Saroli, an Apothecary at San Vittore del Lazio, in Italy, claiming that such was the case, and attaching considerable importance to the fact.

During an epidemic of diphtheria, Mr. Saroli had treated one hundred and fifty-two cases, by my method, and out of this number he had lost only two, infants at the breast, whom it was impossible to treat with any degree of thoroughness. He afterwards subjected the mixture employed to a chemical analysis, and found a new combination, which he designates as an Alcoholic Iodo-phenyl-hydrate, implying a true chemical combination of the iodine and the carbolic acid. He declares one of the characteristics of this combination to be its peculiar relations to Ozone. A piece of paper saturated therewith is a much more sensitive ozonometer than Schonbeins Iodide of Potassium and Starch.

Whether carbolic acid in this chemical union with iodine acts any more powerfully on the diphtheritic membrane (especially in the way of destroying and preventing the return of the fungi) than a simple solution of the acid alone, I do not pretend to say, and I leave the solution of the question to those gentlemen whose positions in public institutions give them better opportunities to test such matters than can be enjoyed in private practice.

As regards my method of application, it has been from the beginning, quite energetic. By means of a pretty good sized camel's hair brush, I apply the lotion, thoroughly and copiously, to all parts of the mouth and throat covered by the exudation. In severe cases this application is repeated every three hours, in lighter ones, two or three times a day. In children that are old enough, I order frequent gargling (say every quarter of an hour, or half an hour) with a solution of thirty drops of the mixture to a tea-cup full of water. I never forcibly remove any portions of the exudation, but only bring away such pieces thereof as spontaneously cling to the brush. I have therefore never, even after the throwing off of the diphtheritic coat, encountered any considerable losses of substance, though occasionally ulcers of the size of a pea have remained for a few days close to the uvula. In case that the exudation spreads into the naso-pharyngeal space, with a discharge from the nose, I introduce the lotion on a small brush through the nostrils. I have never but once met with any paralysis of the pharynx following the treatment, and that yielded promptly to the application of the constant current. The high fever, generally present, was combatted with Digitalis.

At the expiration of from two to ten days the exudation begins to diminish in area and to be cast off, the healthy mucous membrane appearing underneath it.

The definite, uniform course of the disease, under this treatment, has led me to conclude that, at the beginning of the attack, the local process is the main thing and the accompanying fever only secondary; also that, by the early and energetic use of this treatment, the exudation may, in the majority of cases, be controlled, and constitutional infection prevented.

The solution, as directed above, is of a reddish-brown color, at first turbid, but after standing for some time becomes clear and transparent.—*Kansas City Med. Journal.*

